# “All about communication" - a perspective on our public involvement practices in the Networked Data Lab in Grampian.

**DOI:** 10.23889/ijpds.v7i3.1912

**Published:** 2022-08-25

**Authors:** Magdalena Rzewuska, Sharon Gordon, William Ball, Jessica Butler, Allison Poppel, ACHDS PPIE Group

**Affiliations:** 1 Health Services Research Unit, University of Aberdeen; 2 Aberdeen Centre for Health Data Science, University of Aberdeen

## Objectives

The Networked Data Lab (NDL) in Grampian has been embedding evidence-informed practices of working in active partnership with the public in producing insights on population health care. We aim to describe and explain our experiences by reflecting on this journey.

## Approach

We co-developed a National Institute for Health Research (NIHR) guidance-based patient and public involvement and engagement (PPIE) framework with the funder, designed local activities and developed processes and materials for each activity. We created, trained, and involved our PPIE group and delivered tailored online interactions with people that represent service users. We applied best practice principles: institutional buy-in, clarity of purpose, two-way communication, accessibility to broader public, continuity, be designed to impact and evaluated. Existing guidance proposes how to involve the public, whereas a communication theory can explain what 'really happens' during involvement and why. We documented and engaged in reflective assessments of our practice and applied a communication perspective to describe it inductively.

## Results

We use two communication concepts. We exchange information/ideas with the public (Transmission Model) to help decide what we will do, clarify issues, and solve problems. This approach focuses on what's said/meant/heard, expressing it clearly and through the best channel. The PPIE lead's role in that is to minimise interferences in transmission. We also explore what evolves from communication between people (Coordinated Management of Meanings theory) – the nature of our relationships, who is included, what views and experiences individuals bring into our conversations, what they want from it, how it impacts them, and how cooperation/conflicts emerge. This second communication concept is a step outside traditional power relations. The PPIE lead's role in that is to shape a dialogue where multiple perspectives are heard and productive tension is maintained.

## Conclusion

Specific methods (such as NIHR guidance) are only one component of effective involvement practice. Communication is the primary social process underpinning public involvement. Understanding communication may help to guide how to use it as a creative force behind co-production in health data science.

